# Cholecystectomy outcomes after endoscopic sphincterotomy in patients with choledocholithiasis: a meta-analysis

**DOI:** 10.1186/s12876-020-01376-y

**Published:** 2020-07-17

**Authors:** Jie Xu, Chuang Yang

**Affiliations:** 1 North Sichuan Medical College, Nanchong, Sichuan Province China; 2 Hepatobiliary Surgery, The third Hospital of Mianyang·Sichuan Mental Health Center, Mianyang, Sichuan Province China

**Keywords:** Cholecystectomy, Choledocholithiasis, Sphincterotomy, Meta-analysis

## Abstract

**Background:**

Endoscopic sphincterotomy (ES) is the standard treatment for common bile duct stones. The reported findings regarding complications, such as biliary pancreatitis and cholangitis, differ between cholecystectomy after ES. The purpose of this study is to compare cholecystectomy outcomes after endoscopic treatment of common bile duct stones whether or not the incidence of recurrent pancreatitis and cholangitis is reduced, especially in high-risk patients.

**Methods:**

We analyzed 8 studies, including 7 randomized controlled trials retrieved from the PubMed (1990–2019), Embase (1990–2019), and Cochrane (1990–2019) databases for trials comparing the two strategies for treatment of gallstones after ES. A related study on gallbladder removal after ES was acquired, followed by analysis of each group using RevMan. Risk ratios (RRs) were calculated for categorical variables and differences in means were calculated for continuous variables.

**Results:**

We retrieved a total of 8 studies, including seven randomized controlled trials and one retrospective study. A total of 12,717 patients were included in the study (4922 in the early cholecystectomy group and 7795 in the gallbladder in situ group). During the follow-up period, 41 patients had pancreatitis after ES in the cholecystectomy group and 177 patients in the wait-and-see group. The incidence of pancreatitis in the cholecystectomy group was significantly reduced (RR, 0.38; 95% CI, 0.27–0.53; *P* < 0.00001; I^2^ = 0%). The incidence of cholangitis and jaundice in the cholecystectomy group was also less than the preserved gallbladder group (RR, 0.31; 95% CI, 0.26–0.38; *P* < 0.00001; I^2^ = 0%). There was no significant difference in mortality between the two groups (RR, 0.73; 95% CI, 0.52–1.02; *P* = 0.07; I^2^ = 14%). There was a significant difference in cholecystitis and biliary colic (RR, 0.28; 95% CI, 0.24–0.32; *P* < 0.00001; I^2^ = 17%).

**Conclusion:**

Early cholecystectomy after removal of common bile duct stones can effectively reduce biliary complications. This is still true for high-risk patients and has no significant effect on the mortality of patients. Laparoscopic cholecystectomy is recommended after ES.

## Background

Gallstone disease is a common medical condition worldwide, with 10–20% of patients with choledocholithiasis developing biliary pancreatitis and cholangitis [1, 2]. Treatment of common bile duct stones and gallstones includes open biliary exploration + cholecystectomy, laparoscopic cholecystectomy + biliary exploration, and endoscopic sphincterotomy (ES) + laparoscopic cholecystectomy [3, 4]. Classen first reported ES for the treatment of bile duct stones in 1974 [5]. ES subsequently became the mainstream treatment for common bile duct stones [6, 7].

For the treatment of common bile duct stones, multiple guidelines recommend cholecystectomy after endoscopic removal of bile duct stones [8, 9]. Some patients choose to preserve the gallbladder in situ because of the associated risk or an unwillingness to undergo cholecystectomy again. Escourur first reported a case of gallbladder retention after ES in 1984 [10]. Approximately 22% of patients who retain the gallbladder developed biliary complications [11]. It has been suggested that the gallbladder in situ after endoscopic treatment, and the risk of biliary symptoms in patients with asymptomatic stones appears to be equal, without the need to remove the gallbladder [12]. Compared with the preservation of the gallbladder, cholecystectomy after removal of the bile duct stones, although increased hospital stay, can reduce the recurrence rate of postoperative biliary complications [11–13].

Our goal was to perform this systematic assessment of randomized controlled trials and large sample retrospective studies to elucidate the difference between cholecystectomy and gallbladder in situ after endoscopic treatment in patients with common bile duct stones.

## Methods

### Data sources and search strategy

Using the PubMed, EMBASE, and Cochrane library databases, we searched for all published studies on the endoscopic treatment of common bile duct stones with gallstones from January 1990 to April 2019.The following MeSH, Emtree, and keyword search terms were used in various combinations: “cholelithiasis” OR “bile duct stone” OR “common bile duct stone” OR “gallstone” OR “bile lithiasis” OR “bile lithogenicity” OR “biliary calculi” OR “biliary lithiasis” OR “biliary tract calculus” OR “cholecystolithiasis” OR “gallbladder calculus” OR “gallbladder stone” OR “choledochal calculus” OR “choledocholithiasis” OR “choledochus calculus” OR “choledochus stone” OR “common bile duct calculi” OR “common bile duct calculus” OR “common biliary duct stone” OR “ductus choledochus stone” AND “sphincterotomy” OR “endoscopic sphincterotomy” OR “sphincterotomy” AND “cholecystectomy” OR “gallbladder resection” OR “gall bladder resection”.

### Inclusion and exclusion criteria

Two authors (JX and CY) searched for original studies using predetermined inclusion criteria. Patients who met the following requirements were included in the study: 1) successful endoscopic removal of common bile duct stones; 2) removal of the gallbladder or retention of the gallbladder; 3) ≥ 18 years of age; and 4) randomized trials or studies with samples greater than 500. The exclusion criteria were as follows: case reports; and duplicate reports. Two researchers scrutinized the titles and abstracts of all identified articles, first excluding unrelated studies, and then reading the full text to further rule out unqualified studies.

### Data extraction and quality assessment

Two commentators independently extracted data according to standardized extraction forms. The main extracts included author, year of publication, country in which the study was conducted, age and gender of the patient, type of endoscopic technique, intervention (wait-and-see or cholecystectomy), and the design and follow-up of the trial, including complications, such as cholecystitis, biliary colic, cholangitis, pancreatitis, and jaundice.

This systematic review and meta-analysis were carried out strictly in accordance with the guidelines of the preferred reporting items for systematic reviews and meta-analyses [14]. The review protocol is included in the Supplementary data. We used the Cochrane tool for assessing the risk of bias for quality assessment of individual studies. This tool assesses the presence of selection bias by evaluating the methods of randomization and allocation concealment; specifically, performance and detection of bias was determined by checking for blinding of personnel and outcome assessment and attrition, and reporting bias was determined by evaluating for incomplete and selective data reporting. Quality assessment was independently carried out by two reviewers (JX and CY), and differences of opinion were resolved by discussion to reach a consensus.

### Data synthesis and statistical analysis

Data analysis was performed in RevManAnalysis5.3. Relative risk (RR) and 95% confidence intervals (CIs) were calculated with software (RevManAnalysis5.3) using the number of events and the number of patients in both groups.

Our primary outcome of interest was acute pancreatitis between the two groups. The secondary outcomes included the difference in mortality, biliary colic, cholecystitis, cholangitis, recurrent jaundice, major adverse events, and length of hospital stay. These secondary outcomes were pooled using a fixed effects model in the meta-analysis. To estimate statistical heterogeneity, we used the I^2^ statistic, where an I^2^ > 50% indicated significant heterogeneity. The fixed effects model was used in the meta-analysis when the heterogeneity was < 50%, while an I^2^ > 50% was used in the random effects model.

The exclusion method was used for the sensitivity analysis, but only when the results were relevant. For primary outcome measures, evidence of publication bias and other biases were assessed based on a regression analysis of the funnel plot asymmetry. The source of heterogeneity was assessed by sensitivity and subgroup analyses. The first subgroup analysis was based on risk assessment of patients (high-, low-, and unclear-risk). High-risk patients were defined as one of the following: American Society of Anesthesiologists IV or V; age > 65 years; and multiple co-morbidities like cardiac disease. Two trials could not be assessed for risk. The low-risk group excluded high-risk patients. The unclear-risk group included high- and low-risk patients.

## Results

### Study selection and characteristics

**Fig. 1:**
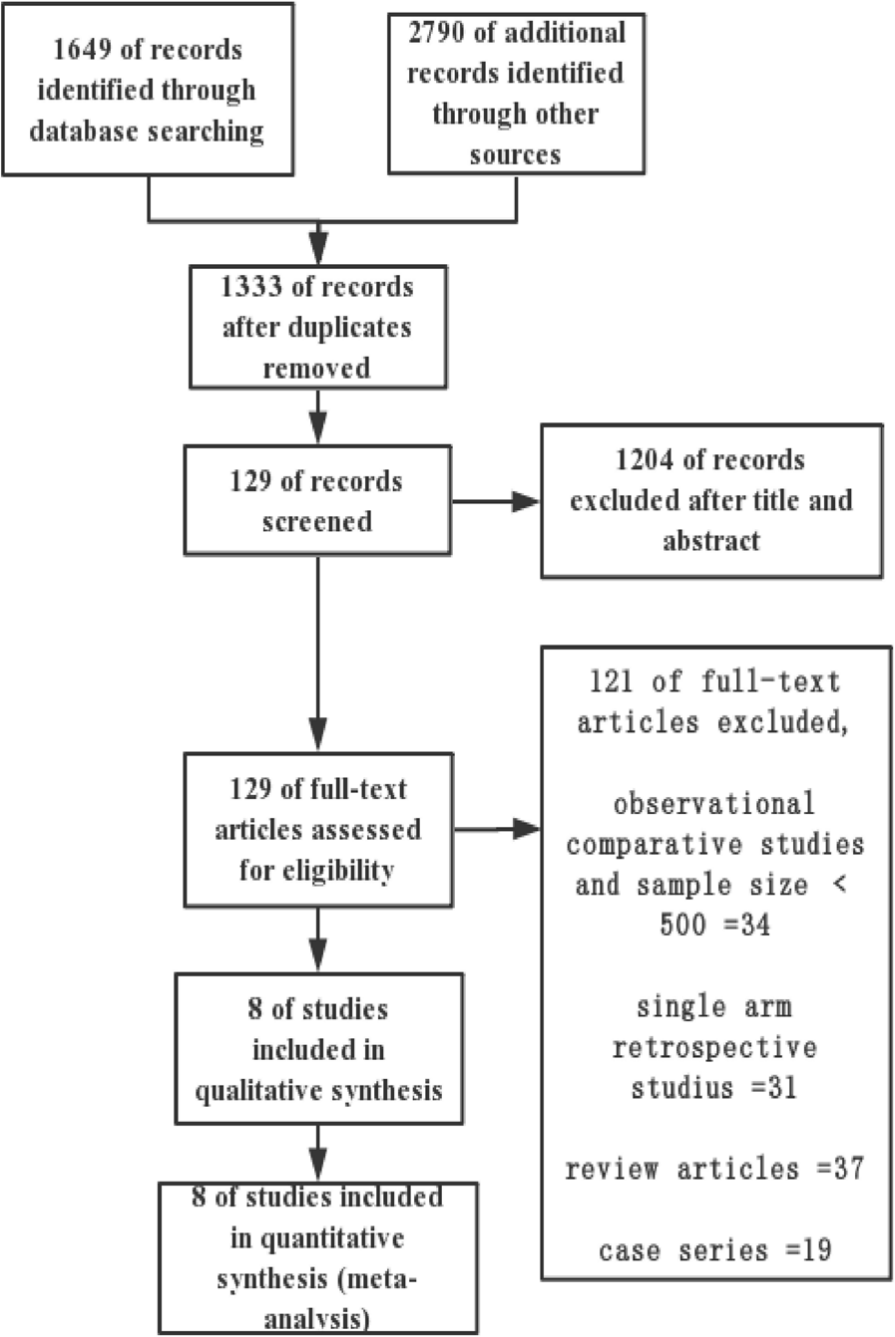
Flow diagram of studies included in the meta-analysis


A flow diagram of our systematic review is shown in Fig. 1. The initial search identified 4439 potential studies. After a review of titles and abstracts, 1204 studies were rejected due to data duplication, irrelevant purpose, or comments. One hundred twenty-nine articles were searched for more detailed assessments and full-text reviews. Based on randomization and the sample size of the retrospective study, eight articles were finalized. A total of 12,717 subjects were included, including seven randomized trials and one retrospective study; the data were collected from 1995 to 2018 [15–22]. The baseline characteristics of the included studies are presented in Table 1.

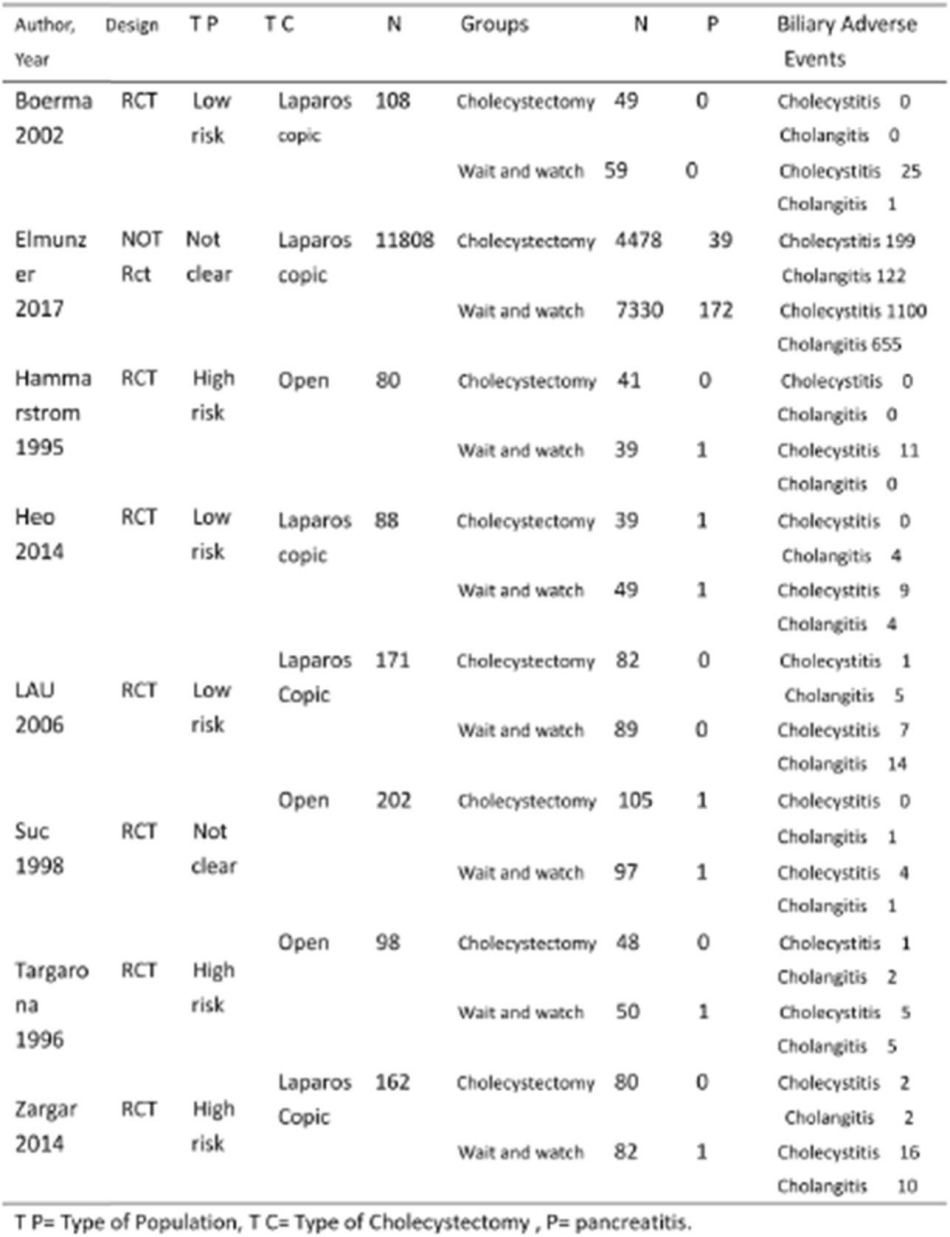

**Table 1:**
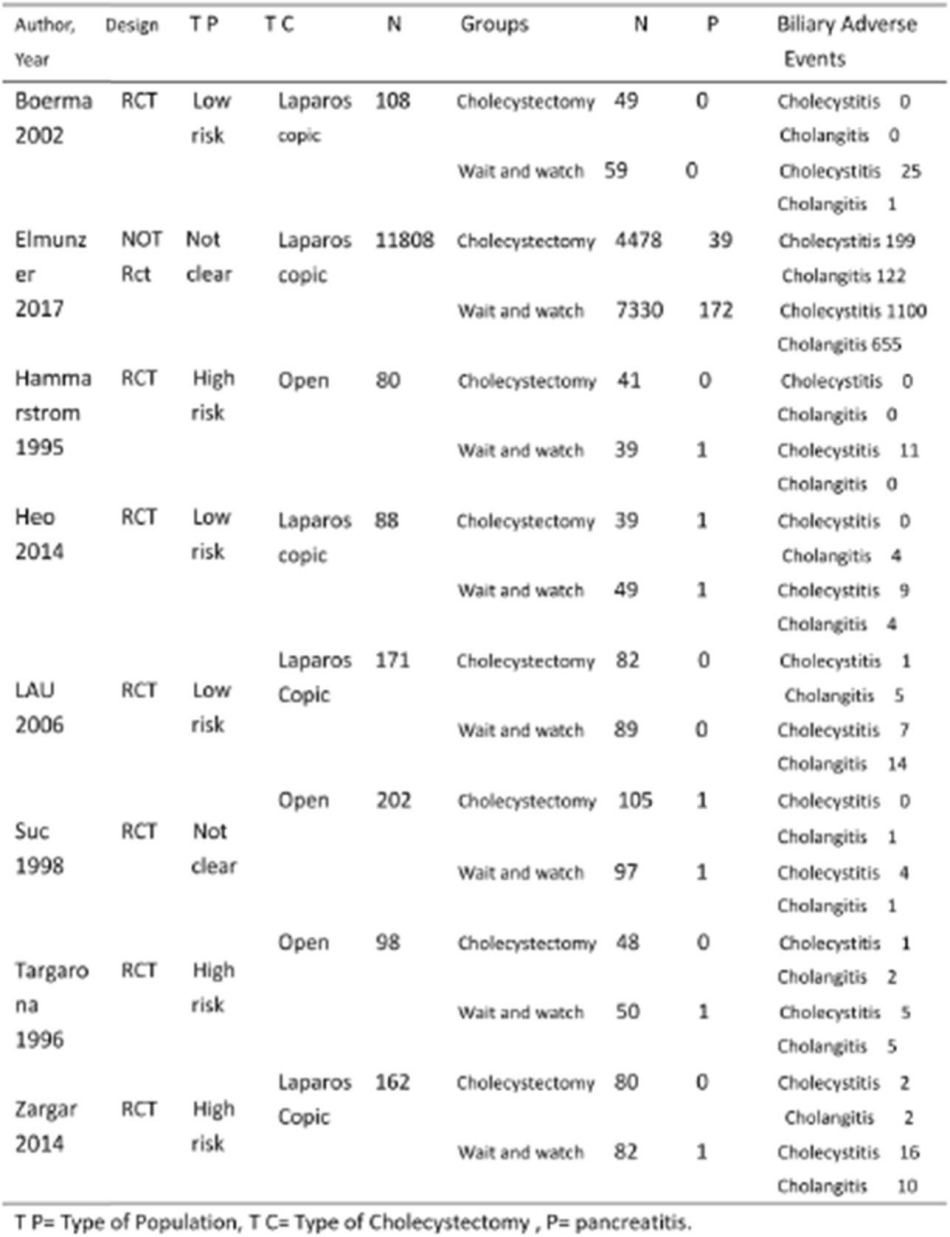
Baseline characteristics of the included studies

### Risk of bias and quality of evidence

**Fig. 2:**
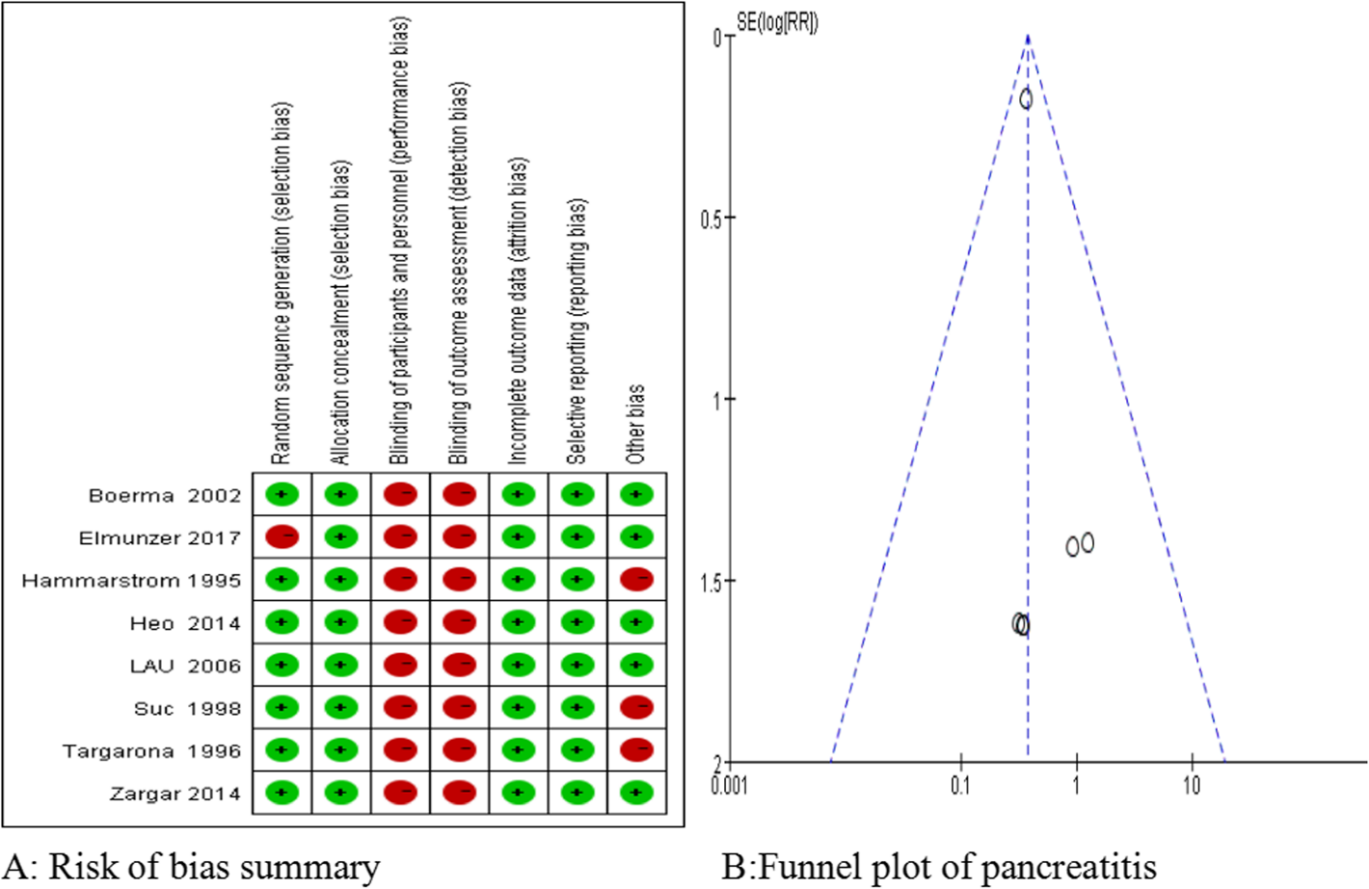
Risk of bias summary and funnel plot of pancreatitis. **a**: Risk of bias summary: review authors’ judgments about each risk of bias item for each included study. **b**: Funnel plot of pancreatitis

The bias risk of the included studies was critically assessed using the Cochrane Collaboration risk of bias tool. Seven studies were randomized, and one retrospective study was a large sample study. All of the studies were unblinded and the remaining bias was low, thus the evidence was considered reliable. The bias assessment of each methodologic component from the eligible studies is shown in Fig. 2.

### Meta-analysis

**Fig. 3:**
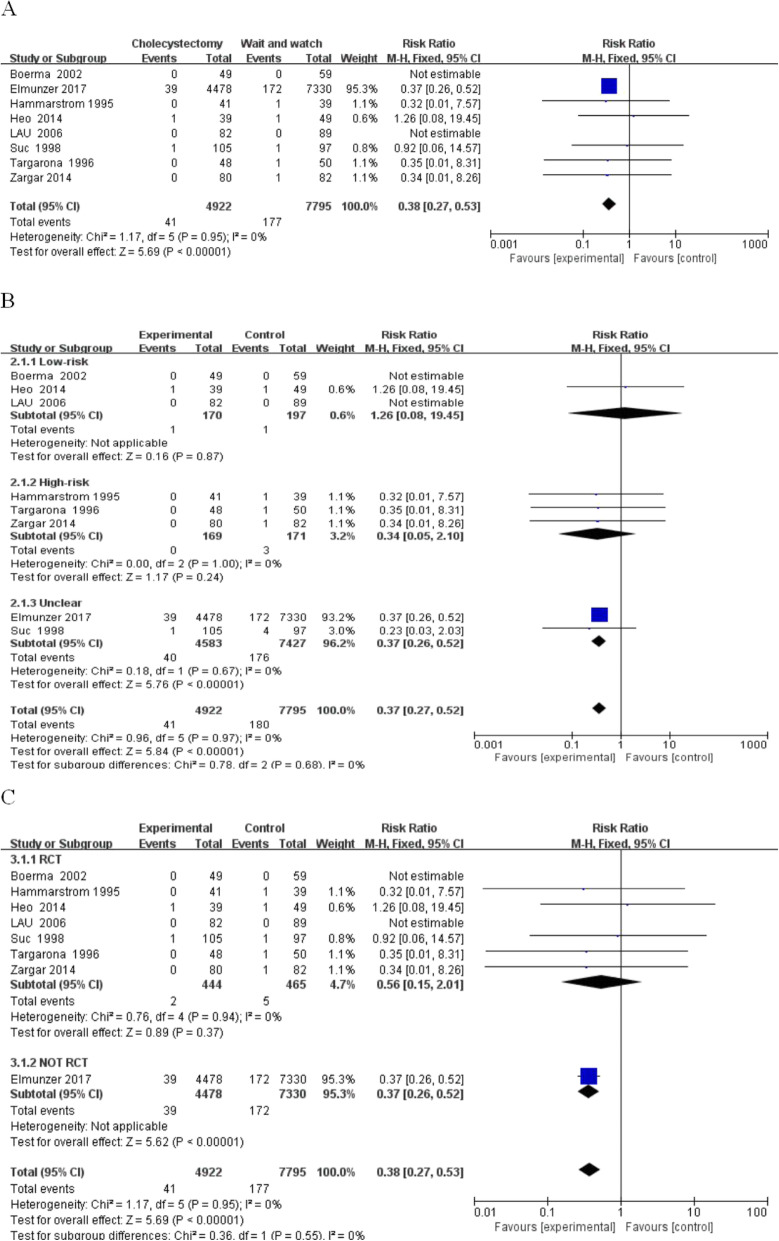
Pancreatitis between the wait-and-see group and cholecystectmy group. **a** Pancreatitis in two group people; **b** subgroup analysis of pancreatitis in low and high-risk patients; **c**: subgroup analysis of pancreatitis in rct and not rct research

(1) Recurrent acute pancreatitis: There was a total of 12,717 patients in 8 studies (4922 in the cholecystectomy group). Pancreatitis occurred in 41 patients (0.83%) after ES in the cholecystectomy group and 177 patients (2.27%) in the wait-and-see group (Fig. 3). Recurrent pancreatitis was compared between the resection and gallbladder in situ group (RR, 0.38; 95% CI, 0.27–0.53; *P* < 0.00001; I^2^ = 0%).

The first subgroup analysis was based on a comparison of the high and low risk groups: low-risk group (RR, 1.26; 95% CI, 0.08–19.45; *P* = 0.87); high-risk group (RR, 0.34; 95% CI, 0.05–2.1; *P* = 0.24; I^2^ = 0%); and unclear-risk-group (RR, 0.37; 95% CI, 0.26–0.52; *P* < 0.00001; I^2^ = 0%). Another subgroup analysis revealed the following: randomized research group (RR, 0.56; 95% CI, 0.15–2.01; *P* < 0.37; I^2^ = 0%); and retrospective study group (RR, 0.37; 95% CI, 0.26–0.52; P < 0.00001).

There is no I^2^ statistic for the low-risk and retrospective groups because two trials did not have acute pancreatitis in the gallbladder in situ or cholecystectomy groups and one trial in the retrospective group. Randomized trials were compared with retrospective studies. The incidence of pancreatitis in the resection group was 0.45 and 0.83%, respectively. The incidence of pancreatitis in the gallbladder in situ group was 1.07 and 2.27%, respectively. Finally, the sensitivity analysis was performed using the exclusion method one-by-one. There was no significant difference in pancreatitis between the small samples. The large sample study was different from the small sample statistical analysis because the incidence of pancreatitis was low and the difference in large samples was more stable and more apparent. Small sample results were unstable, which causes deviation in the statistical analysis due to individual cases.

(2) Cholangitis and recurrent jaundice: Among the 12,717 patients evaluated the rate of the incidence of cholangitis in the resection group and the gallbladder in situ group (RR, 0.31; 95% CI, 0.26–0.38; *P* < 0.00001; I^2^ = 0%).

**Fig. 4:**
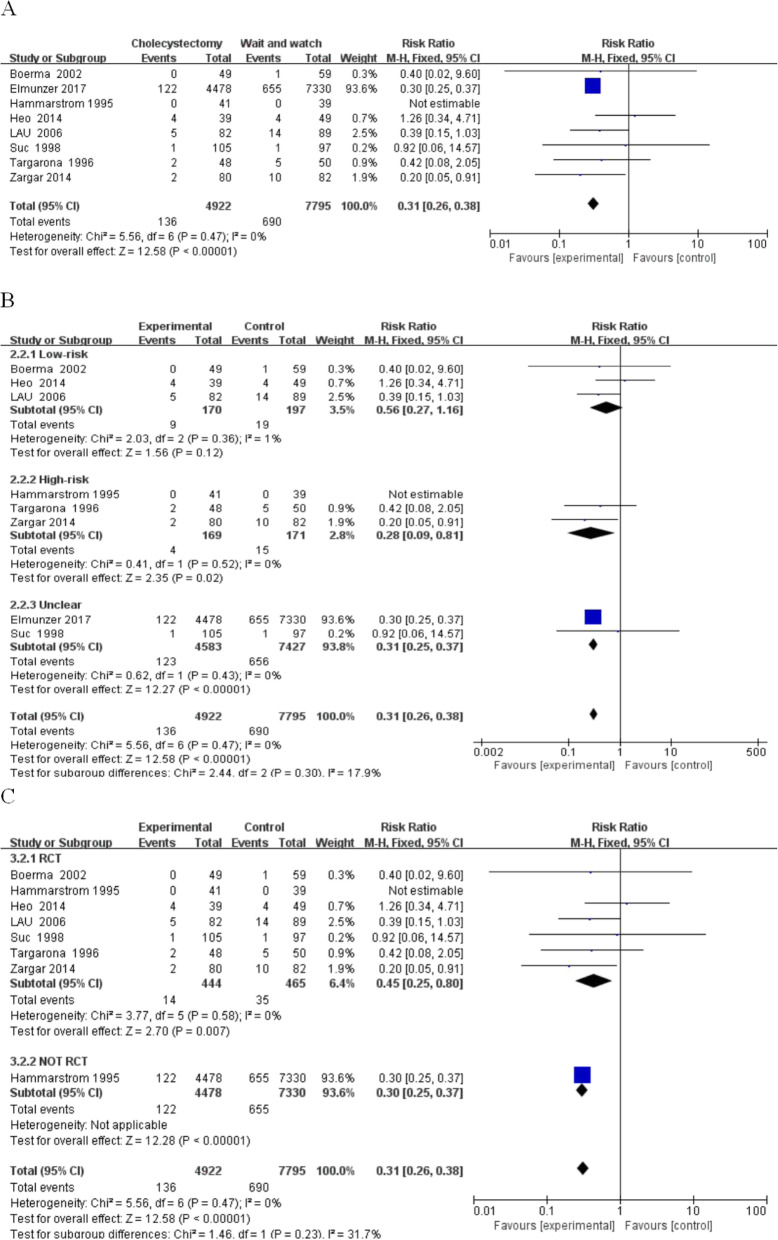
Cholangitis between the wait-and-see group and cholecystectmy group. **a** Cholangitis in two group people; **b** Subgroup analysis of cholangitis in low and high-risk patients; **c** Subgroup analysis of cholangitis in rct and not rct research

The incidence of cholangitis and jaundice in the gallbladder group was less than the preserved gallbladder group (Fig. 4). The first subgroup analysis showed the following: in low-risk group (RR, 0.56; 95% CI, 0.27–1.16; *P* = 0.12; I^2^ = 1%), high-risk group (RR, 0.28; 95% CI, 0.09–0.81; *P* = 0.02; I^2^ = 0%), unclear-risk-group (RR, 0.31; 95% CI, 0.25–0.37; *P* < 0.00001; I^2^ = 0%). There was no significant difference in the low-risk group; however, there was a difference between the high-risk and unidentified-risk-groups. Another subgroup analysis revealed the following: Randomized experimental group (RR, 0.45; 95% CI, 0.25–0.8; *P* = 0.007; I^2^ = 0%), retrospective study group (RR, 0.31; 95% CI, 0.26–0.38; P < 0.00001). In the randomized and retrospective groups, the proportion of cholangitis and recurrent jaundice in the final cholecystectomy group was lower than the conservative group.

**Fig. 5:**
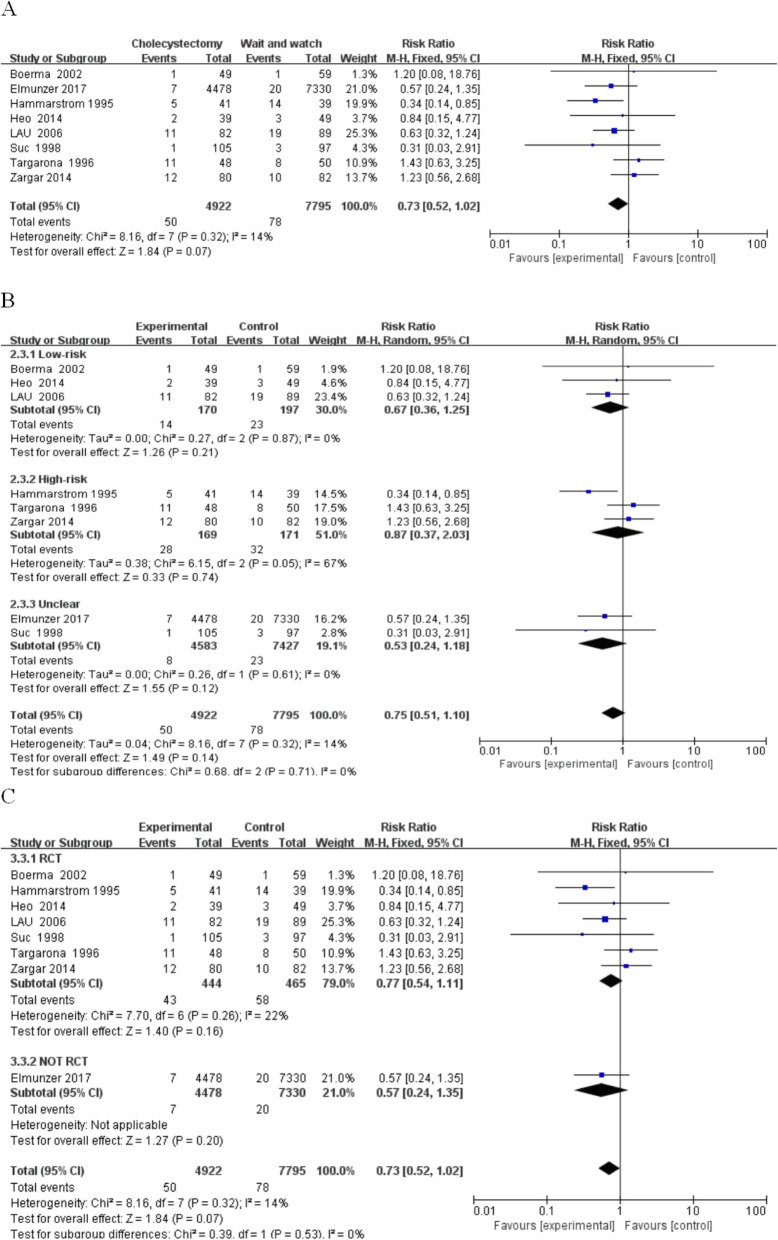
Deaths between the wait-and-see group and cholecystectmy group. **a** Deaths in two group people; **b** Subgroup analysis of deaths in low and high-risk patients; **c** Subgroup analysis of deaths in rct and not rct research

(3) Mortality: A comparison of mortality between the resection and conservative groups in the 8 studies (RR, 0.73; 95% CI, 0.52–1.02; *P* = 0.07; I^2^ = 14%) revealed that there was no significant difference in mortality between the two groups (Fig. 5). Subgroup analysis based on risk grading was as follows: in the low-risk group (RR, 0.67; 95% CI, 0.36–1.25; *P* = 0.21; I^2^ = 0%), high-risk group (RR, 0.87; 95% CI, 0.37–2.03; *P* = 0.74; I^2^ = 67%), and unclear-risk-group (RR, 0.53; 95% CI, 0.24–1.18; *P* = 0.12; I^2^ = 0%).

All groups showed that there was no significant difference in mortality between the two groups. The high-risk group had higher heterogeneity and adopted a random effect pattern that was mainly caused by the Hammarstrom study using the excavation method. It may be because the sample size of this study was small, and the data have certain contingency.

(4) Biliary colic and cholecystitis: Cholecystitis and biliary colic were compared in the post-operative resection and wait-and-see groups (RR, 0.28; 95% CI, 0.24–0.32; *P* < 0.00001; I^2^ = 17%).

**Fig. 6:**
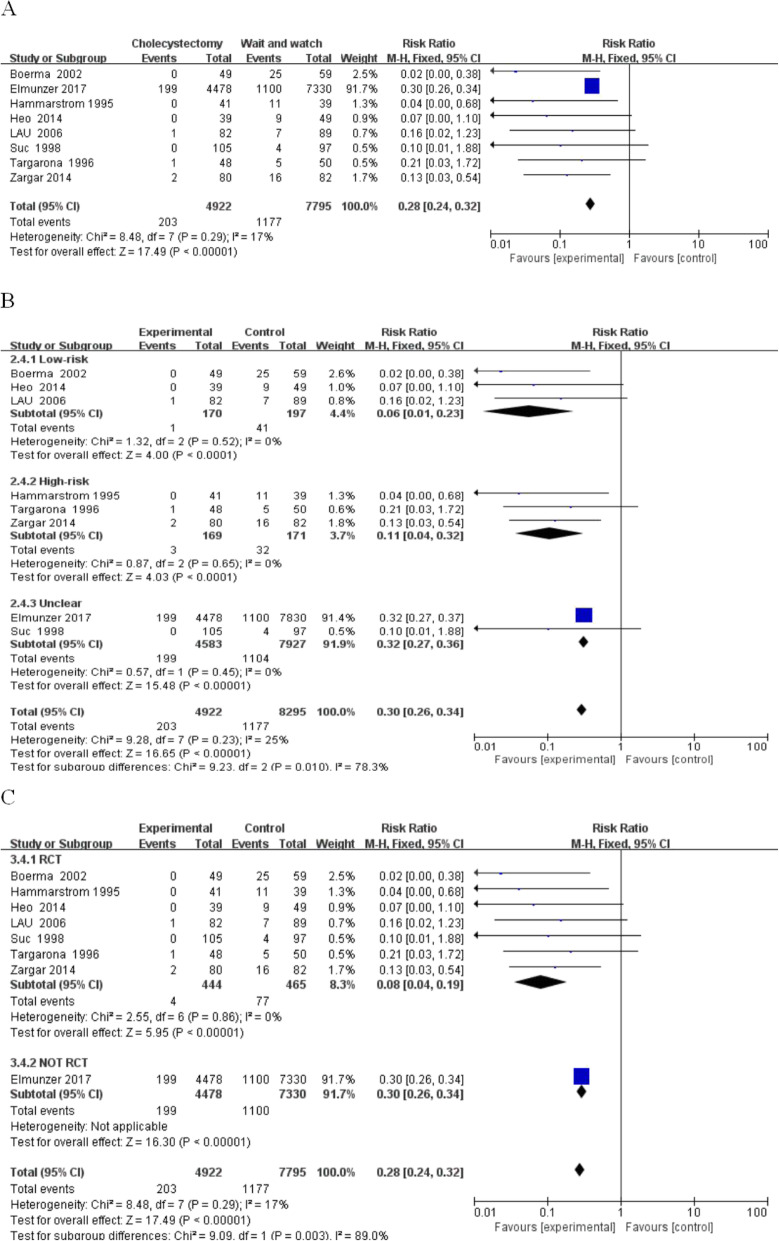
Biliary colic and cholecystitis between the wait-and-see group and cholecystectmy group. **a** Biliary colic and cholecystitis in two group people; **b** Subgroup analysis of biliary colic and cholecystitis in low and high-risk patients; **c** Subgroup analysis of biliary colic and cholecystitis in rct and not rct research

The comparison in the subgroup analysis showed the following (Fig. 6): low-risk group (RR, 0.06; 95% CI, 0.01–0.23; *P* < 0.0001; I^2^ = 0%); high-risk group (RR, 0.11; 95% CI, 0.04–0.32; P < 0.0001; I^2^ = 0%); and unclear-risk-group (RR, 0.32; 95% CI, 0.27–0.36; *P* < 0.00001; I^2^ = 0%). Subgroup analysis based on research typing revealed the following: Randomized research (RR, 0.08; 95% CI, 0.04–0.19; P < 0.00001; I^2^ = 0%) and retrospective study groups (RR, 0.30; 95% CI, 0.26–0.34; P < 0.00001).

In all analyses, the incidence of cholecystitis and biliary colic in the early resection of the gallbladder group was significantly lower than the gallbladder group, and the heterogeneity was lower in all subgroups.

### Publishing bias

The funnel plot does not show substantial asymmetry (Fig. 2).

## Discussion

This study analyzed the effect of cholecystectomy after ES. Specifically, whether or not the recurrence of pancreatitis and cholangitis can be reduced after cholecystectomy was compared. The main conclusions of our review were that the gallbladder in situ *group* compared with the cholecystectomy group resulted in an increased incidence of pancreatitis, more patients with biliary pain and cholecystitis, and more patients with recurrent jaundice and cholangitis, but there was no significant difference in mortality between the two groups. Even in the high-risk group, active cholecystectomy can effectively reduce the occurrence of biliary complications.

This study combined the advantages of both randomized and retrospective studies. The retrospective study showed the difference in recurrence of pancreatitis due to the large amount of data in addition to an insufficient number of randomized studies. Randomized studies increase the objectivity of retrospective studies. The combination of holistic and subgroup analyses compensates for the sample size gap between randomized and retrospective studies. Through holistic and subgroup analyses, we comprehensively analyzed the conclusions of randomized and retrospective studies, which affirmed our findings and increased the credibility of the conclusions. The major limitations of our review were as follows: one of the studies was a retrospective study. Retrospective research reduces the credibility of the evidence and increases the risk of bias; the sample size of the randomized study was small, the sample size gap between the retrospective study was large; and the number of randomized studies was small.

Some studies have suggested that cholecystectomy after endoscopic treatment of bile duct stones can effectively reduce the occurrence of biliary complications, especially the incidence of cholecystitis and cholangitis [23, 24]; however, most studies have concluded that there is no significant difference in the recurrence rate of pancreatitis. To determine whether or not there was a difference in the recurrence of pancreatitis, this analysis has added a large sample study [25, 26]. The amount of retrospective data included in this study was large, and the results of randomized studies did not have an effective impact. The final analysis results were determined by retrospective studies. Based on the subgroup analysis, the grouping of large samples was different from the grouping results of small samples.

The statistical results were not different in the small sample study, but there were differences in large samples because there were few randomized studies, there were limited experimental data in the randomized studies, and as the incidence of pancreatitis was low, there was more contingency, which resulted in unstable results. In contrast, due to the large number of retrospective studies, the difference was stable, more representative of pancreatitis in both groups, and statistically significant.

Large sample studies amplify the difference in the incidence of pancreatitis in both groups, and the larger sample results are more reliable than small samples. From the two groups of studies, the incidence of pancreatitis in the cholecystectomy group was < 1%, and the incidence of the gallbladder in situ group was < 2.5%. Therefore, it is believed that the early cholecystectomy after endoscopic removal of bile duct stones reduces the incidence of pancreatitis.

The articles included in this manuscript had no specific reports on the size and quantity of gallstones. Grouping according to gallstones and re-analysis may yield different conclusions. Single large stones have a lower risk of recurrence of biliary pancreatitis and cholangitis compared to small stones and biliary stones < 5 mm in size, but the incidence of cholecystitis in older patients increases with age, although recurrent pancreatitis is not increased, the risk of cholecystitis increases, and thus it is still recommended to remove the gallbladder after ES [27–29].

## Conclusions

This analysis supports the finding that prophylactic cholecystectomy after endoscopic treatment of common bile duct stones can effectively reduce complications, such as pancreatitis, cholangitis, and cholecystitis, but there was no significant difference in mortality from all causes in both groups. Even in the high-risk group (ASA grade III–IV patients), it is recommended that patients undergo surgery to remove the gallstones after removal of common bile duct stones, thus reducing the possibility of re-operation.

## Supplementary information

**Additional file 1.** *Date, *Results, *The main result included: Recurrent acute pancreatitis, Cholangitis and recurrent jaundice, Mortality and Biliary colic and cholecystitis.

## Data Availability

All data generated or analysed during this study are included in this published article [and its supplementary information files]. Competing interests. The authors have no relevant affiliations or financial involvement with any organization or entity with a financial interest in or financial conflict with the subject matter or materials discussed in the manuscript. This includes employment, consultancies, honoraria, stock ownership or options, expert testimony, grants or patents received or pending, or royalties.

## Abbreviations

CI: Confidence interval
ES: Endoscopic sphincterotomy
RR: Risk ratio
ASA: American Society of Anesthesiologists
RCT: Randomized Controlled Trial
